# Temporal relationship between dysthymia and temporomandibular disorder: a population-based matched case-control study in Taiwan

**DOI:** 10.1186/s12903-017-0343-z

**Published:** 2017-02-01

**Authors:** Shang-Lun Lin, Shang-Liang Wu, Shun-Yao Ko, Ching-Yu Yen, Wei-Fan Chiang, Jung-Wu Yang

**Affiliations:** 1 Department of Psychiatry, Kaohsiung Armed Forces General Hospital, Kaohsiung, Taiwan; 2 Graduate Institute of Medical Science, College of Health Science, Chang Jung Christian University, Tainan, Taiwan; 3 School of Medicine, Griffith University, Gold Coast, Australia; 4 School of Dentistry, Taipei Medical University, Taipei, Taiwan; 5 Department of Oral and Maxillofacial Surgery, Chi-Mei Medical Center, Yongkang, Tainan Taiwan; 6 Department of Oral and Maxillofacial Surgery, Chi-Mei Medical Center, Liouying, Tainan Taiwan; 7 School of Dentistry, National Yang-Ming University, Taipei, Taiwan; 8 Department of Oral and Maxillofacial Surgery, Tainan Sin Lau Hospital, the Presbyterian Church in Taiwan, Tainan, Taiwan; 9 Yuan Yuan Dental Federation, Tainan, Taiwan; 10 701 No. 57, Sec. 1, East Gate Road, East Dist., Tainan City,, Taiwan, R.O.C.

## Abstract

**Background:**

Numerous studies have reported a relationship between depression and temporomandibular disorders (TMD), but the conclusions remain undefined. The aim of this article was to examine the temporal relationship between depression and TMD.

**Methods:**

In this retrospective matched case-control study, we recruited all samples from a randomsample sub-dataset of one million insured individuals for the year 2005 (Longitudinal Health Insurance Database (LHID2005)). All beneficiaries were enrolled in the National Health Insurance (NHI) programme in Taiwan. We used propensity scoring and matched the case and control groups (1:1) by ten confounding factors to detect the effect of different types of depression on TMD.

**Results:**

The positive correlative factors of TMD included the total number of times medical advice was sought for an unspecified anomaly of jaw size plus malocclusion (TTSMA-JS, *p* = 0.045), the total number of times medical advice was sought for an anxiety state (TTSMA-AS, *p* = 0.000), and the total number of times medical advice was sought for a panic disorder (TTSMA-P, *p* = 0.009). Dysthymia (synonymous with chronic depression) had an effect on TMD. The odds ratio (OR) of dysthymia for TMD measured by multiple logistic regression was 1.91 (*p* = 0.008) after adjusting for demographic factors, psychiatric comorbidities, and maxillofacial confounders.

**Conclusions:**

This study demonstrated the established temporal relationship between dysthymia and TMD. The inclusion of a psychiatrist on the TMD management team is appropriate.

## Background

Temporomandibular disorders (TMD) are a significant public health problem globally. A meta-analysis study reported that 30% of randomly selected subjects showed a perceived dysfunction, and 44% demonstrated a clinically assessed dysfunction of their temporomandibular joint (TMJ) pain with/without joint sound [1]. TMD has been considered a multifaceted and complex disease process [2]. Most TMD patients sought treatment because of pain involving the pre-auricular region, jaw, head, and neck [3]. The presence of pain elsewhere in the body, female gender, and pre-existing depressive symptoms are related to the onset of TMD [4]. Pain-related TMD may, therefore, have an influence on the quality of life with disability, psychosocial and behavioural consequences [5]. The clinical ICD-9 codes for TMD only focus on the physical domain of pain, joint mobility, and disc location. The diagnostic criterion for TMD (DC/TMD) includes psychosocial status evaluation (Axis II) as a necessary component [6].

Depression has been increasing over time, and it is one of the leading causes of disease-related disability worldwide [7]. A large cross-sectional epidemiological study of Portuguese college students reported that 61.4% of the students with the symptom of TMD also had signs of anxiety or depression [8]. Various studies have indicated that painful temporomandibular disorders are associated with high levels of depression [9–11]. Furthermore, these conditions are often bi-directional and co-existent [9, 10]. However, these studies were unable to prove a temporal relationship between depression and TMD. Moreover, a population-based cohort study design could not determine whether depression was a source or consequence of TMD [2]; thus, this study was not supportive of evidence-based medicine. Previous TMD studies seldom mentioned the three main types of depression: (1) a major depressive disorder is a severe episodic depression for at least 2 weeks; (2)dysthymia is persistent depression for at least 2 years; and (3) a depressive disorder, not elsewhere classified, is characterized by a minor episode (different than a major depressive disorder) and a shorter course than dysthymia.

The propensity score, which is an adequate method for population allocation, can minimize the differences between groups. Propensity score analysis can reduce the bias of an observational study and adjust for the main confounders [12, 13]. No current TMD case-control studies have used the propensity score for a large population and thereby maximizing the validity.

The purpose of this study was to demonstrate the temporal relationship between depression and TMD utilizing a case-control study design. We assumed that depression is one of the crucial risk factors for TMD. Depressive patients who seek professional help for mental health care and have a willingness to seek medical resources are more likely to be diagnosed with and treated for TMD compared with the general population [14].

## Methods

### Data resources

The National Health Insurance system, which is a source of study data, has provided insurance coverage for more than 98% of the 23 million Taiwanese people and has contracted with more than 93% of medical institutions since 1996. The National Health Research Institute (NHRI) administered all medical claims information recorded from the contracted health care facilities. We retrieved all sampled subjects from the Longitudinal Health Insurance Database (LHID2005). The LHID2005 includes all of the original medical claims and registration files for one million enrollees in the NHI programme. The one million random-sample enrollees in the LHID2005 were taken from all insured persons registered in the 2005 registry of beneficiaries (*N* = 23.72 million) [15]. This dataset contained the registry of medical facilities, orders of inpatients and outpatients, dental services, and prescriptions linked to anonymous identifications. Thousands of published SCI papers had proven the high validity of NHI data [16, 17]. Because all patient identifications were released to the public for research purposes, the LHID was omitted from full review by the Institutional Review Board in Taiwan. We still obtained an ethical certificate (protocol number SLH919-104-007) from the Ethics Committee of Tainan Sin Lau Hospital, the Presbyterian Church in Taiwan.

### Study samples

**Fig. 1:**
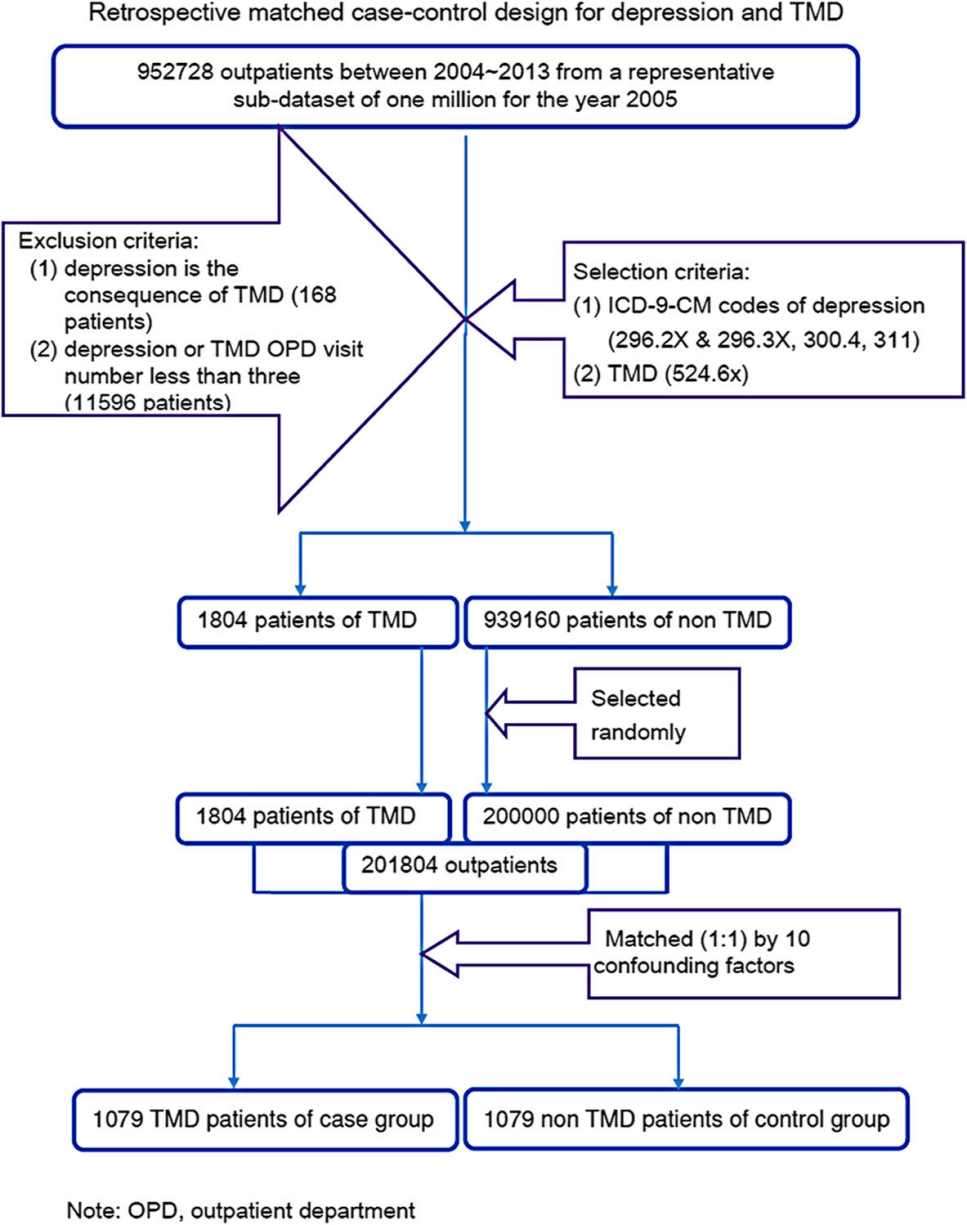
Flowchart of sample selection

In selecting samples for analysis in this retrospective matched case-control study, we first recruited 952,728 outpatients between 2004 and 2013 from a representative onemillion sub-datasets. We selected the samples by ICD-9-CM (International Classification of Diseases, 9th revision, Clinical Modification) codes for TMD (ICD-9-CM codes 524.61, 524.60, 524.63, 524.62, and 524.69) and depression (ICD-9-CM codes 296.2X, 300.4, 296.3X, and 311). In our study design, the independent variables were three general types of depressive disorders: depressive disorders not elsewhere classified (ICD-9-CM code 311), dysthymia (ICD-9-CM code 300.4), and major depressive disorders (ICD-9-CM codes 296.3X and 296.2X). To avoid a selection bias of TMD diagnoses and depression, we only included those patients who had at least three clinical records for TMD and depression during the follow-up period after the index date. This study excluded the following cases: (1)TMD that occurred before the onset of depression (168 patients) and (2) a total of OPD visits for depression or TMD less than three (11,596 patients). A total of 13,568 TMD patients and 939,160 non-TMD patients were included in the initial pool of patients via the recruiting process. From this initial pool, 1804 TMD patients with qualified criterions and 200,000 non-TMD random-selected patients were analyzed for the study. The matching protocol (1:1) for the patients (201,804) had 10 confounding variables, including monthly income, age, sex, and the total number of times seeking medical advice (TTSMA) for the following illnesses: mandible fracture (ICD-9-CM codes 802.2 and 802.3), anxiety state (ICD-9-CM code 300.00), unspecified anomaly of jaw size plus malocclusion (ICD-9-CM codes 524.0–524.5), panic disorder (ICD-9-CM code 300.01), generalized anxiety disorder (ICD-9-CM code 300.02), obsessive compulsive disorders (ICD-9-CM code 300.03), and psychiatric diseases except the above-mentioned disorders (ICD-9-CM codes 290–319). A total of 1079 outpatients were allocated to the case group and the control group (Fig. 1). The follow-up period for the case and control groups was between January 1, 2004, and December 31, 2013. For the case group without depression, the starting point was the first ambulatory care visit (including outpatient departments of hospitals or clinics); for the case group with depression, the starting point was a time of the first diagnosis of depression. For the case unit, the end point was the time of the first diagnosis of TMD.

### Statistical analysis

We used G-power 3.1.2 to determine the sample size (z-tests, logistic regression, and two tails). With an odds ratio = 1.407, X distribution = binomial, *R* ^2^ = 0.2, α = 0.05, and power = 0.8, it was calculated that the total required sample size for this study was 1920 [18].

The study generated the propensity score of each case by choosing depression (yes/no) as the dependent variable and the ten confounders as independent variables in “predicted values, probabilities” of logistic regression. The matching of the propensity score ensured that the probabilities of depression were exactly equal in the TMD group and the control group.

We compared the ten confounders (no coded cases of obsessive compulsive disorders (ICD-9-CM code 300.03)) of demographic variables and comorbidities between TMD and non-TMD patients using the propensity score. Multiple logistic regression analysis with the odds ratio (OR) was used to estimate the risk of TMD associated with depression after adjusting for confounders. We executed all analyses using SPSS statistical software (version 20 for Windows; IBM; New York, USA).

## Results

The gender distribution comparison of patients between the two groups was identical. There were more females than males in both the TMD and non-TMD groups (71.3% versus 28.7%, respectively).

Table 1 shows the comparisons of the continuous variables of confounders between case and control groups. There were no differences in monthly income (*p* = 0.899), age (*p* = 0.431), TTSMA-MF (*p* = 0.160), TTSMA-GA (*p* = 0.299), and TTSMA-PD (*p* = 0.109). There were differences in TTSMA-JS(*p* = 0.045), TTSMA-AS (*p* = 0.000), and TTSMA-P (*p* = 0.009) between the two groups.

**Table 1:** Continuous variables of confounders between two groups

variables	mean	sd	Min.	Max.	F	*p*
MI					0.02	0.899
control	26,422	24,287	0	182,000		
case	26,556	24,420	0	182,000		
Age					0.62	0.431
control	38.93	17.59	1.00	88.00		
case	39.53	17.81	1.00	92.00		
TTSMA-MF					1.97	0.160
control	0.00	0.00	0.00	0.00		
case	0.04	0.98	0.00	31.00		
TTSMA-JS					4.03	0.045
control	0.09	0.45	0.00	7.00		
case	0.14	0.83	0.00	15.00		
TTSMA-AS					32.43	0.000
control	1.23	6.95	0.00	132.00		
case	3.92	13.88	0.00	145.00		
TTSMA-P					6.93	0.009
control	0.05	1.04	0.00	31.00		
case	0.32	3.20	0.00	55.00		
TTSMA-GA					1.08	0.299
control	0.28	4.63	0.00	123.00		
case	0.46	3.26	0.00	45.00		
TTSMA-PD					2.57	0.109
control	1.68	12.19	0.00	218.00		
case	2.52	12.13	0.00	165.00		

Depression: 296.2, 296.3, 300.4, 311 (ICD-9 codes). MI: monthly income. TTSMA-MF: the total number of times seeking medical advice for mandible fracture. TTSMA-JS: the total number of times seeking medical advice for the unspecified anomaly of jaw size. TTSMA-AS: the total number of times seeking medical advice for anxiety state. TTSMA-P: the total number of times seeking medical advice for panic disorder. TTSMA-GA: the total number of times seeking medical advice for generalized anxiety disorder. TTSMA-PD: the total number of times seeking medical advice for psychiatric diseases (ICD-9 codes: 290.X-319) except depression

In Table 2, multiple logistic regression analysis shows that the risk of TMD was significantly greater in the dysthymia (ICD-9-CM code 300.4) (OR 1.91, *p* = 0.008) after adjusting for the ten confounders. Another confounder, TTSMA for anxiety state (TTSMA-AS), demonstrated a significant positive correlation with TMD(OR 1.03, *p* = 0.000).

**Table 2:** Odds ratio of temporomandibular disorder in depressions adjusted for confounders in multiple logistic regression

variables	OR(95% CI)
T296(yes/no)	1.02(0.37 ~ 1.67)
T300.4(yes/no)	1.91(1.44 ~ 2.38)
T311(yes/no)	0.68(-0.06 ~ 1.42)
MI	1(-0.96 ~ 2.96)
Age	1(-0.96 ~ 2.96)
TTSMA-MF	93(-3809.01 ~ 3995.01)
TTSMA-JS	1.13(-0.34 ~ 2.6)
TTSMA-AS	1.03(1.01 ~ 1.05)
TTSMA-P	1.06(-0.51 ~ 2.63)
TTSMA-GA	1(-0.23 ~ 2.23)
TTSMA-PD	1(-0.08 ~ 2.08)
Sex(Male/Female)	1.06(0.86 ~ 1.26)

T296: Ever seeking medical advice for major depressive disorder. T300.4: Ever seeking medical advice for dysthymia. T311: Ever seeking medical advice for Depressive disorder, not elsewhere classified. MI: monthly income. TTSMA-MF: the total number of times seeking medical advice for mandible fracture. TTSMA-JS: the total number of times seeking medical advice for the unspecified anomaly of jaw size. TTSMA-AS: the total number of times seeking medical advice for anxiety state. TTSMA-P: the total number of times seeking medical advice for panic disorder. TTSMA-GA: the total number of times seeking medical advice for generalized anxiety disorder. TTSMA-PD: the total number of times seeking medical advice for psychiatric diseases (ICD-9 codes: 290.X-319) except depression

The mean follow-up time was 4.71 ± 2.85 years (after matching), the minimum follow-up period was 0 years, and the maximum follow-up period was 10 years for both groups.

## Discussion

Temporomandibular disorders are associated with high levels of depression [8, 9, 19]. However, studies of cross-sectional models only present a possible association and not a temporal relationship. Although Macfarlane et al. found in their case-control study that people with PDS (pain dysfunction syndrome) were characterized by high levels of psychological distress [10], they could not clarify whether depression occurred prior to the onset of TMD or as a consequence of TMD.

Our study was similar to previous research showing that females had a higher rate of TMD than males [2, 20], and depressive patients had an increased risk of TMD [2]. The odds ratio of dysthymia for TMD was 1.91, which was lower than that of the previous case-control study (OR = 2.5) [10]. The reason for this finding might be because other subtypes of depression and the ten confounding factors interfered with the effect of dysthymia. As confounding factors, both mandible fracture and an unspecified anomaly of jaw size with malocclusion or dentofacial deformity are not in the causal pathway of depression to TMD, but they are associated with depression [21, 22] and TMD [23–25].

The virtues of our study, which has great internal validity, are as follows: our study included a 10-year follow-up period; the definition of depression and TMD were based on diagnoses by specialists and required at least three visits; TMD occurred prior to depression was excluded; and case and control groups were strictly matched (1:1) by propensity score. Moreover, we adjusted for demographic factors, psychiatric comorbidities of depression and maxillofacial confounding factors in the multiple logistic regression analysis. All of these factors showed minimal differences between two groups and powerfully increased our internal validity. The minimal and maximal ranges of all variables represent most of the Taiwanese population and ensure external validity. Therefore, the character of TMD in our study was supportive of more validity. Additionally, we investigated dysthymia’s unique effect on TMD, results of which supported that pain and mood disorders might share similar neurobiological mechanisms and neuroanatomical constructs, especially chronic mood disorder [26]. Depressed patients are inclined to have symptoms of pain and consume more health resources than non-depressed patients. Therefore, dysthymia (a synonym for chronic depression) patients with temporomandibular pain tend to acquire a diagnosis of TMD when seeking typical medical services. Our findings are consistent with the fact that dysthymia patients had the most statistically significant pain symptoms [26]. Successful depression treatment might reduce the pain of TMD [27].

There are some limitations to our study. First, orthodontic treatment and orthognathic surgery do not appear in the National Health Insurance program, and patients still have to pay without assistance from the insurance programme. Therefore, we could not provide the ICD-9-CM codes from the insurance program, with which our confounding factors of TMD would have been more complete. Second, clinically common symptoms such as bruxism, which might highly affect TMD, could not be coded using ICD-9-CM. Additionally, ICD-9-CM couldnot clearly code the following TMDs: condylysis/idiopathic condylar resorption, synovial chondromatosis, osteochondritis dissecans, osteonecrosis of the temporomandibular joint, and systematic arthritis with involvement of the temporomandibular joint. Third, our study design did not allow us to present the temporal effects of key confounders, such as the anxiety state. Further studies with different independent variables might help clarify these issues. Fourth, to reduce the differences between the case and control groups, we sacrificed 725 cases, which may have reduced our external validity. Finally, we found it difficult to categorize the subtypes of TMD found in the database using ICD-9-CM codes; therefore, evaluating the impact of depression on subtypes of TMD is an arduous undertaking.

## Conclusions

This study demonstrated that dysthymia had a temporal relationship with TMD. The results suggest that a psychological evaluation should be part of the primary management of TMD, and these patients should receive a referral to a psychiatrist as needed. Further cohort studies are required to evaluate issues such as whether TMD has a causal effect on depression, how various types of depression influence different subtypes of TMD, and whether maxillofacial problems (such as mandible fracture, and the anomaly of jaw size with malocclusion) can be causative risk factors for TMD.
